# Emerging insights on internet gaming disorder: Conceptual and measurement issues

**DOI:** 10.1016/j.abrep.2019.100242

**Published:** 2019-12-11

**Authors:** Halley M. Pontes, Vasileios Stavropoulos, Mark D. Griffiths

**Affiliations:** a Division of Psychology, School of Medicine, University of Tasmania, Launceston, Australia; b The International Cyberpsychology and Addictions Research Laboratory (iCARL), University of Tasmania, Launceston, Australia; c School of Psychology, College of Health and Biomedicine, Victoria University, Melbourne, Australia; d Psychology Department, Nottingham Trent University, Nottingham, United Kingdom

Video gaming has become one of the most prevalent forms of leisure activity in today’s societies. The latest data from the Entertainment Software Association (ESA) reported that approximately 65% of all American adults play video games regularly and that approximately 75% of households have at least one gamer (Entertainment Software Association, 2019). Video gaming is widely accessible and the most commonly used gaming devices among adult gamers in the United States of America (USA) are smartphones (60%), personal computers (52%), and dedicated game consoles (49%) (Entertainment Software Association, 2019). A similar trend has been reported in other countries. For example, in Australia, the Interactive Games and Entertainment Association (IGEA) recently reported that 67% of all Australians play video games and that 97% of households with children have computer games (Interactive Games & Entertainment Association, 2017). For the majority of gamers, the activity leads to positive and beneficial outcomes including psychomotor, cognitive, therapeutic, and educational benefits (Granic et al., 2013, Griffiths, 2019, Nuyens et al., 2017). In general, gamers report that playing has a positive impact on their lives, with nearly 80% of all American adult gamers reporting that games provide mental stimulation, relaxation, and stress relief (Entertainment Software Association, 2019). Similarly, the majority of Australian gamers report that gaming helps them improve their thinking skills (84%), dexterity (78%), and manage pain (59%).

Although the great majority of gamers report experiencing similar positive outcomes, the extant literature has systematically reported detrimental and harmful effects stemming from excessive gaming, particularly in relation to disordered gaming (Burleigh et al., 2019, Nuyens et al., 2019, Şalvarlı and Griffiths, 2019, Stavropoulos et al., 2019). The consequences of excessive and addictive gaming have been researched for over 35 years (e.g., Harry, 1983, Ross et al., 1982) and the psychiatric importance of this phenomenon has been steadily increasing since 2013.

In light of the latest conceptual and diagnostic advances in the field, the American Psychiatric Association (APA) included ‘Internet Gaming Disorder’ (IGD) in the fifth revision of the *Diagnostic and Statistical Manual for Mental Disorders* (DSM-5) (American Psychiatric Association, 2013) as a tentative addictive disorder warranting further research. Furthermore, the APA has proposed a diagnostic framework defining IGD as a condition characterized by disordered gaming behavior leading to significant clinical impairments within a period of 12 months as indicated by the endorsement of five out of nine following diagnostic criteria. These criteria include: (i) preoccupation with games (‘*preoccupation*’); (ii) withdrawal symptoms when gaming is taken away (‘*withdrawal*’); (iii) tolerance, resulting in the need to spend increasing amounts of time gaming (‘*tolerance*’); (iv) unsuccessful attempts to control gaming activities (‘*loss of control*’ and *‘relapse’*); (v) loss of interest in previous hobbies and pastime activities as a result of, and with the exception of, gaming (‘*giving up other activities*’); (vi) continued excessive gaming behavior despite the knowledge of psychosocial problems (‘*continuation*’); (vii) deceiving family members, therapists, or others regarding the amount of gaming (‘*deception*’); (viii) gaming to escape or cope with negative mood states (‘*escape*’ and *‘mood modification’*); and (ix) jeopardizing or losing significant relationships, jobs, or education or career opportunities due to gaming (‘*negative consequences*’) (American Psychiatric Association, 2013).

More recently, additional developments in the field culminated in the long-awaited formal recognition of ‘Gaming Disorder’ (GD) as a behavioral addiction by the World Health Organization (WHO) in May 2019 (Griffiths and Pontes, 2019, Pontes and Griffiths, 2019). Accordingly, GD is characterized by a pattern of online and/or offline gaming behaviors that is persistent and indicated by the following clinical criteria: impaired control over gaming (i.e., onset, frequency, intensity, duration, termination, context – ‘*loss of control*’); increasing priority given to gaming to the extent that it takes precedence over other life interests and daily activities (‘*giving up other activities*’); and continuation or escalation of gaming despite the occurrence of negative consequences (‘*continuation*’). Additionally, these symptoms must occur within a 12-month timeframe and the behavior pattern must be of sufficient severity and lead to significant impairments (i.e., personal, family, social, educational, occupational) across important areas of life (‘*negative consequences*’) (World Health Organization, 2019).

Given the public health relevance of this emerging phenomenon, researchers must continue to carry out high-quality research to further improve the current understanding of GD and the way in which it negatively impacts the life of a minority of gamers. To this end, previous robust epidemiological research has reported prevalence rates of GD usually below 5% among nationally representative samples (Pontes, 2018). It is clear that the research community is taking seriously the issue of GD as timely special issues dedicated to different aspects of GD have been recently published on a number of refereed journals. These included recent special issues focusing on investigating “Internet Gaming Disorder: A Pathway Towards Assessment Consensus” (Stavropoulos, Gomez, et al., 2019) and the “Neural Mechanisms Underlying Internet Gaming Disorder” (Zhang & Brand, 2018).

With this in mind, the present special issue sought to contribute to the many ongoing debates in the field of GD (see Ferguson et al., 2019, Ferguson and Colwell, 2019, Griffiths et al., 2016, Schimmenti and Starcevic, 2019, van Rooij et al., 2018) and to expand the knowledge base of this condition by encouraging additional relevant research focusing on providing novel insights to help tackle conceptual and measurement issues surrounding GD. This is a timely challenge given that the two major diagnostic frameworks for this condition are not consistent in the number of criteria needed to be endorsed to diagnose GD. Such inconsistencies led Pontes, Schivinski, Brzozowska-Woś, and Stavropoulos (2019) to suggest that the operationalization for GD according to the APA and WHO frameworks highlight important discrepancies at the clinical level. Furthermore, these authors posited that the WHO framework arguably takes a laxer approach when defining the condition by reducing the number of criteria needed to be endorsed, potentially contributing to over-diagnosis and over-pathologization of GD among gamers. This is a key consideration given that the choice of diagnostic framework itself may negatively affect diagnostic accuracy of GD in terms of specificity and sensitivity, as well as positive and negative predictive values, which are key paraments in clinical assessment. Interestingly, such discrepancies between these two diagnostic frameworks have been found to interfere with the estimation of prevalence rates in GD (Montag et al., 2019), and a recent neuroimaging study found key neurobiological differences associated with the adoption of the two diagnostic frameworks to measure GD (Zhou et al., 2019).

In this context, the studies published within this special issue of *Addictive Behaviors Reports* on GD provide further valuable empirical insights concerning this condition at several levels. Overall, the studies recruited culturally and developmentally diverse samples from several countries, including Italy (Triberti et al., 2018), United Kingdom (Moudiab & Spada, 2019), Poland (Schivinski, Brzozowska-Woś, Buchanan, Griffiths, & Pontes, 2018), USA (Snodgrass et al., 2019, Stavropoulos et al., 2019), Australia (Hu et al., 2019, Scerri et al., 2019, Stavropoulos et al., 2019, Stavropoulos et al., 2019), New Zealand (Hu et al., 2019), and China (Snodgrass et al., 2019), as well as participants from different Europeans countries (Snodgrass et al., 2019). Although all the studies in the special issue recruited participants via online surveys, they used a different range of analytical strategies to provide empirical insights concerning GD. These included performing basic multivariate statistical analyses such as testing multiple linear regression models (Moudiab and Spada, 2019, Snodgrass et al., 2019, Stavropoulos et al., 2019, Stavropoulos et al., 2019, Triberti et al., 2018), latent variable modeling using Exploratory Factor Analysis (EFA), Confirmatory Factor Analysis (CFA), and testing Multiple Indicator, Multiple Cause (MIMIC) models (Schivinski et al., 2018, Snodgrass et al., 2019), Item Response Theory (IRT) (Schivinski et al., 2018), moderation analyses (Stavropoulos et al., 2019, Stavropoulos et al., 2019) and mediation analyses (Hu et al., 2019, Scerri et al., 2019).

In addition to using a relatively diverse array of statistical analyses, the studies published in the special issue yielded important findings. The study by Triberti et al. (2018) investigated the relationship between average time spent playing over day phases (morning, afternoon, night; week days; weekend days), age, and game preferences in relation to GD. The authors concluded that time spent playing over day phases is related to game preferences and age and that GD predicts time spent gaming on mornings. The study by Schivinski et al. (2018) scrutinized the APA diagnostic criteria for IGD in a large-scale survey and found that each IGD criterion presents with distinct clinical weighting when diagnosing this condition, leading the authors to conclude that the nine IGD criteria need to take into account differential clinical weighting in diagnostic practices. The special issue also published the first study ever conducted on the intricacies between the Hikikomori phenomenon and GD (Stavropoulos, Anderson, et al., 2019). This study found that Hikikomori symptoms are usually associated with higher incidence of GD symptoms and living with parents may exacerbate GD in Australian gamers. In another important study, Hu et al. (2019) investigated the interplay between preference for social games, flow levels, and gender in the context of GD. The authors found that exclusive preference for social games is associated with higher levels of flow and GD symptoms while gender did not produce significant effects.

Other research published in the special issue investigated GD in the context of Self-Determination Theory (Deci & Ryan, 1985). This study by Scerri et al. (2019) found that need fulfilment deficits were associated with increased GD symptoms, an association that was mediated by self-esteem and depression but not loneliness. Another study in the special issue investigated psychological motives and maladaptive cognitions among those with GD (Moudiab & Spada, 2019). The authors found that motives associated with coping skills development and maladaptive cognitions associated to overvaluing in-game rewards significantly predicted GD regardless of negative affect and problematic Internet use.

Finally, this special issue of *Addictive Behaviors Reports* included strong contributions from studies examining the cross-cultural effects of GD (Snodgrass et al., 2019, Stavropoulos et al., 2019). In their study, Snodgrass et al. (2019) showed that, in particular, the addictive and problematic dimensions of gaming distress are influenced by culture-specific expressions of achievement motivations, social connection and disconnection, and unique psychosomatic experiences across North American, European, and Chinese gamers. Relatedly, Stavropoulos, Adams, et al. (2019) found that among Australian and American gamers, increased inattention and hyperactivity levels associated with increased GD symptoms among the two samples. The study also found that these associations differed across genders between the two countries because more hyperactive-impulsive and inattentive males in the USA presented higher levels of disordered gaming in comparison to Australian gamers with a similar profile.

Although significant progress appears to have been made in relation to the conceptualization, measurement, understanding, and the treatment of GD behaviors, the extant knowledge in the field necessitates further advancement across several important areas. More specifically, GD manifestations have been often implicated with the psychological attachment developed between gamers and their in-game figure of representation, commonly known as the avatar (Liew et al., 2018, Stavropoulos et al., 2019). Interestingly, the same gamer-avatar association has been associated with potential behavior transference in real-life (e.g., Stavropoulos, Gomez, Mueller, Yucel, & Griffiths, 2019). Therefore, it follows that specific aspects of GD may invite a convergence of the users’ in-game and real-life behaviors, which (depending upon the character adopted online) could be either adaptive or maladaptive. However, further knowledge is required in relation to the specific interplay between offline and online demographics that could accommodate the channeling of in-game behaviors in a gamer’s real life conduct. Such studies are valued as significant because they could offer clinically useful information concerning either real-life disruptive or real-life functional behaviors, precipitated and perpetuated by GD manifestations.

Furthermore, GD appears to be uniquely different when compared to other forms of behavioral addictions because gamers in non-western countries appear to be at a higher risk (American Psychiatric Association, 2013) although this might be a consequence of how different cultures view the activity of gaming more generally (for instance, non-western societies often pathologize any activity that is not educationally- or family-oriented (Griffiths, Kuss, Billieux, & Pontes, 2016). However, gaming-related motivations have not been yet thoroughly examined in the context of cultural differences, such as those related to individualistic-collectivistic or hierarchy reflecting cultural values. This gap in the literature appears significant given the global impact of GD behaviors, as well as their particular effects and consequences in the populations of multicultural societies. Specific knowledge considering the cultural aspects involved with GD would enable the employment of more culturally responsive (and thus resource-effective) prevention and intervention initiatives. Finally, while GD behaviors have been often associated with other forms of addiction (both chemical and behavioral) (both chemical and behavioral; see Burleigh et al., 2019 for a recent review), it is still unclear as to what extent and how ‘addiction hopping’ phenomena occur (e.g., migrating from one form of addiction to another; alternatively known as cross-addiction (Griffiths et al., 2002, Haylett et al., 2004). Such knowledge could significantly advance clinical practice in the field by addressing addictive tendencies holistically and in a more efficient manner. However, regardless of future research directions adopted by scholars internationally, tackling such challenging GD issues requires consistency in regard to the formal diagnostic definitions and measures introduced, as well as appropriately capitalizing on the available empirical and clinical knowledge already accumulated.
